# *In Vivo and In Vitro* Expression of Tenascin by Human Thymic Microenvironmental Cells

**DOI:** 10.1155/1995/64871

**Published:** 1995

**Authors:** Claudia Sondermann Freitas, Jurandy Susana Patricia O'Campo Lyra, Sergio Ranto Dalmau, Wilson Savino

**Affiliations:** 1 Department of Immunology National Cancer Institute Rio de Janeiro Brazil; 2 Laboratory on Thymus Research Department of Immunology Institute Oswaldo Cruz-FIOCRUZ Rio de Janeiro Brazil

**Keywords:** Human thymus, tenascin, extracellular matrix, thymus ontogeny

## Abstract

Increasing evidence reveals that extracellular matrix components can be regarded as a group of mediators in intrathymic T-cell migration and/or differentiation. Yet, little is kown about the expression and putative function of one particular extracellular matrix protein, namely, tenascin in the thymus. Herein we investigated, by means of immunocytochemistry, tenascin expression in normal infant and fetal human thymuses, as well as in cultures of thymic microenvironmental cells.

*In situ*, tenascin distribution is restricted to the medulla and cortico-medullary regions of normal thymuses. This pattern thus differed from that of fibronectin, laminin and type IV collagen, in which subseptal basement membranes were strongly labeled. Interestingly, tenascin did not co-localize with the cytokeratin-defined thymic epithelial cell network. This was in keeping with the *in vitro* data showing that tenascin-bearing cells were nonepithelial (and probably nonfibroblastic) microenvironmental elements.

Studies with fetal thymuses revealed a developmentally regulated expression of tenascin, with a faint but consistent network labeling, in thymic rudiments as early as 12 weeks of gestational age, that progressed to a strong TN expression at 18 weeks of fetal development, which was similar to the distribution pattern observed thereafter, including postnatally.

Our results clearly indicated that tenascin is constitutively expressed in the human thymus, since early stages of thymic ontogeny, and suggest that the cell type responsible for its secretion is a nonepithelial microenvironmental cell.


*Developmental Immunology*, 1995, Vol 4, pp. 139–147
Reprints available directly from the publisher
Photocopying permitted by license only

© 1995 Harwood Academic Publishers GmbH
Printed in Singapore

### *In Vivo* and *In Vitro* Expression of Tenascin by Human Thymic Microenvironmental Cells

CLAUDIA SONDERMANN FREITAS[†], JURANDY SUSANA PATRICIA O'CAMPO LYRA,[‡] SERGIO RANTO DALMAU,[†] and WILSON SAVINO[*‡]

[†]*Department of Immunology, National Cancer Institute, Rio de Janeiro, Brazil*
[‡]*Laboratory on Thymus Research, Department of Immunology, Institute Oswaldo Cruz–FIOCRUZ, Rio de Janeiro, Brazil*

Increasing evidence reveals that extracellular matrix components can be regarded as a group of mediators in intrathymic T-cell migration and/or differentiation. Yet, little is kown about the expression and putative function of one particular extracellular matrix protein, namely, tenascin in the thymus. Herein we investigated, by means of immunocytochemistry, tenascin expression in normal infant and fetal human thymuses, as well as in cultures of thymic microenvironmental cells.

*In situ*, tenascin distribution is restricted to the medulla and cortico-medullary regions of normal thymuses. This pattern thus differed from that of fibronectin, laminin and type IV collagen, in which subseptal basement membranes were strongly labeled. Interestingly, tenascin did not co-localize with the cytokeratin-defined thymic epithelial cell network. This was in keeping with the *in vitro* data showing that tenascin-bearing cells were nonepithelial (and probably nonfibroblastic) microenvironmental elements.

Studies with fetal thymuses revealed a developmentally regulated expression of tenascin, with a faint but consistent network labeling, in thymic rudiments as early as 12 weeks of gestational age, that progressed to a strong TN expression at 18 weeks of fetal development, which was similar to the distribution pattern observed thereafter, including postnatally.

Our results clearly indicated that tenascin is constitutively expressed in the human thymus, since early stages of thymic ontogeny, and suggest that the cell type responsible for its secretion is a nonepithelial microenvironmental cell.

KEYWORDS: Human thymus, tenascin, extracellular matrix, thymus ontogeny.

### INTRODUCTION

Tenascin (TN) is an extracellular matrix glycoprotein involved in mesenchymal-epithelial cell interactions during morphogenetic events including embryonic development (Aufderheide and Ekblom, 1988; Inaguma et al., 1988; Thesleff et al., 1990), tissue repair (Donaldson et al., 1991), inflammation (Mackie et al., 1988), and cancer (Mackie et al., 1987; Inaguma et al., 1988; Soini et al., 1992a). Its expression is temporally regulated in embryonic tissues, although in some adult tissues it remains constitutive (Crossin et al., 1986; Natali et al., 1991; Saga et al., 1991).

Biochemically, TN is a complex structure composed of six covalently linked polypeptides and containing several EGF-life (epidermal growth factor) and fibronectin type III repeats (Jones et al., 1989; Saga et al., 1991). Additionally, isoforms of tenascin have been described, although specific roles for each isoform remain to be determined (Aufderheide and Ekblom, 1988; Chiquet-Ehrismann, 1990; Prieto et al., 1990).

Tenascin modulates cellular adhesion to other extracellular matrix proteins such as fibronectin (Chiquet-Ehrismann et al., 1988; Lotz et al., 1989). It was shown to provide opposite signals, leading either to adhesive or antiadhesive effects, which can be exerted by different sites of TN molecule (Spring et al., 1989).

The immunomodulatory activities ascribed to TN include its ability in regulating transient adhesion of monocytes as well as B and T lymphocytes. In this context, TN was shown to alter T-cell behaviour (Ruegg et al., 1989).

* Corresponding author.

It is noteworthy that TN seems to be constitutively expressed in human and murine lymphoid tissues as spleen and lymph nodes (Liakka and Autio-Harmainen, 1992; Soini et al., 1992b; Ocklind et al., 1993). Particularly regarding the thymus, Natali et al., (1991) did not detect tenascin either in fetal or adult organs, as assessed by immunocytochemistry. However, intrathymic expression of this molecule was suggested, because a 5.5–6 kb TN transcript and the respective protein band have been evidenced in murine developing and adult organs (Saga et al., 1991; Ocklind et al., 1993).

The cell type(s) involved in the putative TN expression within the organ is still a matter of debate. Yet, this is a relevant issue if one consider that intrathymic events of T-cell differentiation are driven by the so-called thymic microenvironment, a tridimensional network composed of distinct cell types, the major one corresponding to thymic epithelial cells (TEC) (see reviews Boyd and Hugo, 1991; van Ewijk, 1991; Boyd et al., 1993).

Lastly, a body of evidence came to implicate extracellular matrix (ECM) components as further mediators of this intrathymic T-cell migration and/or differentiation process (Savino et al., 1993).

Taken together, the data discussed before led us to study TN expression in the human thymus, and to search for which cell type would be responsible for producing this extracellular matrix component in the organ.

### MATERIAL AND METHODS

### Thymus Fragments

Fetal thymuses were obtained from Miguel Couto Hospital (Rio de Janeiro), from apparently normal fetuses, whose development was compatible with the ascribed gestational age, which varied from 12 to 30 weeks. Fragments from normal infant thymuses were obtained from 6 children undergoing surgery for correcting congenital cardiac malformations. Specimens were either immediately frozen for further criostat sectioning or put into sterile Hank's solution for settling primary cultures of microenvironmental cells.

### Antibodies

An anti-TN rabbit serum was kindly provided by Dr. Ruth Chiquet-Ehrismann (Friedrich Miescher Institut, Basel, Switzerland). This reagent was produced after immunizing rabbits with purified chicken TN, and can recognize TN from different species (Chiquet and Fambrough, 1984a, 1984b). Additionally, it was shown not to cross-react with fibronectin (Chiquet and Fambrough, 1984a). A further anti-TN rabbit serum was purchased from Gibco-BRL (Gaithersburg, MD). The anti-TN monooclonal antibody (mAb), clone EB2 was purchased from Biohit (Helsinki, Finland) and was originally described elsewhere (Howeedy et al., 1990).

In addition to anti-TN reagents, antibodies recognizing distinct ECM components were obtained from Institute Pasteur (Centre de Radioanalyse, Lyon). All were polyclonal immunesera produced in rabbits by injection of human plasma fibronectin (FN), type IV collagen (TIV-C), or laminin (LN) purified from the murine EHS (Engelbreth Holm-Swarm) sarcoma (Grimaud et al., 1980). These reagents recognize their corresponding molecules in normal human thymuses (Savino and Berrih, 1984; Berrih et al., 1985). Lastly, a polyclonal anti-cytokeratin (CK) rabbit serum (immunoglobulin fraction of rabbit antiserum to human cytokeratins (Dako Corp., Sta. Barbara, CA) and anti-CK MAbs (clones KL1 and KL4 from Immunotech, Marseille) were used in double-labeling immunofluorescence experiments. These reagents were proved to entirely decorate the human thymic epithelium *in situ* and *in vitro* (Berrih et al., 1984, 1985). The Ig fraction of rabbit antiserum to human factor VIII related antigen (Dako Corp.,) was used to stain the vascular endothelium. Second fluorescent antibodies included fluorescein- or rhodamine-labeled goat anti-rabbit Ig sera (respectively GAR/FITC or GAR/TRITC), a goat anti-mouse Ig (GAM/FITC), and a donkey anti-goat Ig (DAG-FITC). These reagents were purchased from Biosys (Compiegne, France).

### Primary Cultures of Human Thymic Stromal Cells

Human thymus fragments from 5 normal individuals were minced into tiny fragments that were led to adhere onto 25-ml culture flasks during 1 hour. Primary cultures were then settled as previously described (Papiernik et al., 1975; Berrih et al., 1985) using RPMI-1640 medium supplemented with 10% heat-inactivated fetal calf serum, $2 \times 10^{-3}$M L-glutamine, $10^{-3}$M sodium pyruvate, $5 \times 10^{-5}$M 2-mercaptoethanol, 100 IU/ml penicillin, and 100 µg/ml streptomycin (Sigma Chemical Co., St.

Louis). Cultures were kept at 37°C in a humidified atmosphere containing 5% $CO_2$. Monolayers of thymic microenvironmental cells thus obtained were washed in PBS, fixed with methanol for 10 min, and stored at −20°C until being processed for immunocytochemistry.

### Immunocytochemistry

Five-μm thick thymus frozen sections or human thymic stromal cultures, respectively fixed in cold acetone or absolute methanol for 10 min, were processed for immunocytochemistry as detailed before (Berrih et al., 1995). Briefly, specimens were incubated with adequate dilutions of a given anti-TN primary antibody, washed in PBS, and subsequently incubated with the appropriate secondary antibody. In the case of 12-week fetal thymus sections, a third antibody (DAG-FITC) was necessary in order to amplify the positive signal obtained after incubation with the anti-TN polyclonal Ab plus the secondary Ab (GAR-FITC).

In order to determine whether TN distribution pattern *in situ* and *in vitro* co-localized with thymic epithelial cells, specimens were subjected to double-labeling immunofluorescence. In some experiments, the anti-TN serum was revealed with the GAR/TRITC whereas the anti-CK MAb was evidenced with the GAM/FITC. Further specimens were double-labeled using the anti-TN MAb and the anti-CK rabbit serum, respectively, revealed with the GAM/FITC and GAR/TRITC reagents.

Topographic relationships between TN distribution and blood vessels were evaluated by double-labeling using the anti-TN serum and the anti-factor VIII related antigen Ab (herein used as an endothelium marker) that had the GAM/FITC and GAR/TRITC as respective revealing systems.

Lastly, the pattern of tenascin distribution was compared to that of other ECM proteins. For that, the anti-TN mAb was double-labeled with each of the anti-ECM serum, as mentioned before.

### RESULTS

### *In Situ* Distribution of Tenascin in Normal Human Thymuses

The presence of tenascin in normal infant thymus sections was consistently detected and exhibited the same pattern, independently of the anti-TN reagent applied. Tenascin immunoreactivity was restricted to the medulla and cortico-medullary junction of the thymic lobules.

This contrasted with the negative pattern observed at the border and within connective tissue septae, as well as in typical cortical areas (Fig. 1).

This distribution pattern thus differed from that described for basement membrane proteins such as fibronectin, laminin, and type IV collagen, in which subseptal basement membranes were strongly labeled (Berrih et al., 1985). In fact, we confirmed such difference by performing double-labeling immunofluorescence in which TN was detected simultaneously with other ECM proteins, including fibronectin, laminin, and type IV collagen (Fig. 2).

Because TN-labeling adjacently to blood vessels was apparent, we also performed dual immunofluororescence for simultaneous detection of TN and vascular endothelia. We noticed that TN labeling was rather restricted to adventitial layers of blood vessels, whereas endothelial cells, evidenced with the anti-factor VIII mAb, remained negative (not shown).

The further relevant question concerning intrathymic tenascin expression referred to the cell type responsible for its production in normal conditions. In this respect, double-labeling immunofluorescence using anti-TN and anti-CK reagents revealed that *in situ*, TN distribution did not co-localize with the CK-defined thymic epithelial cell

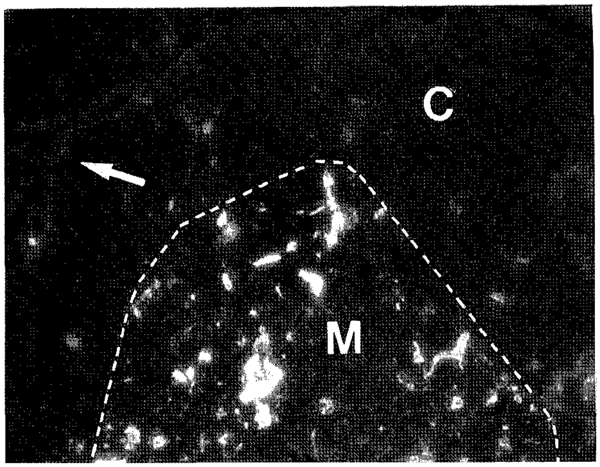

FIGURE 1. Restricted *in situ* distribution of tenascin in normal human thymus as immunohistochemically revealed with the anti-tenascin monoclonal antibody. Dashed line represents the cortico-medullary limits, whereas a perilobular septum is indicated with the arrow. M: medulla; C: cortex. Magnification: ×150.

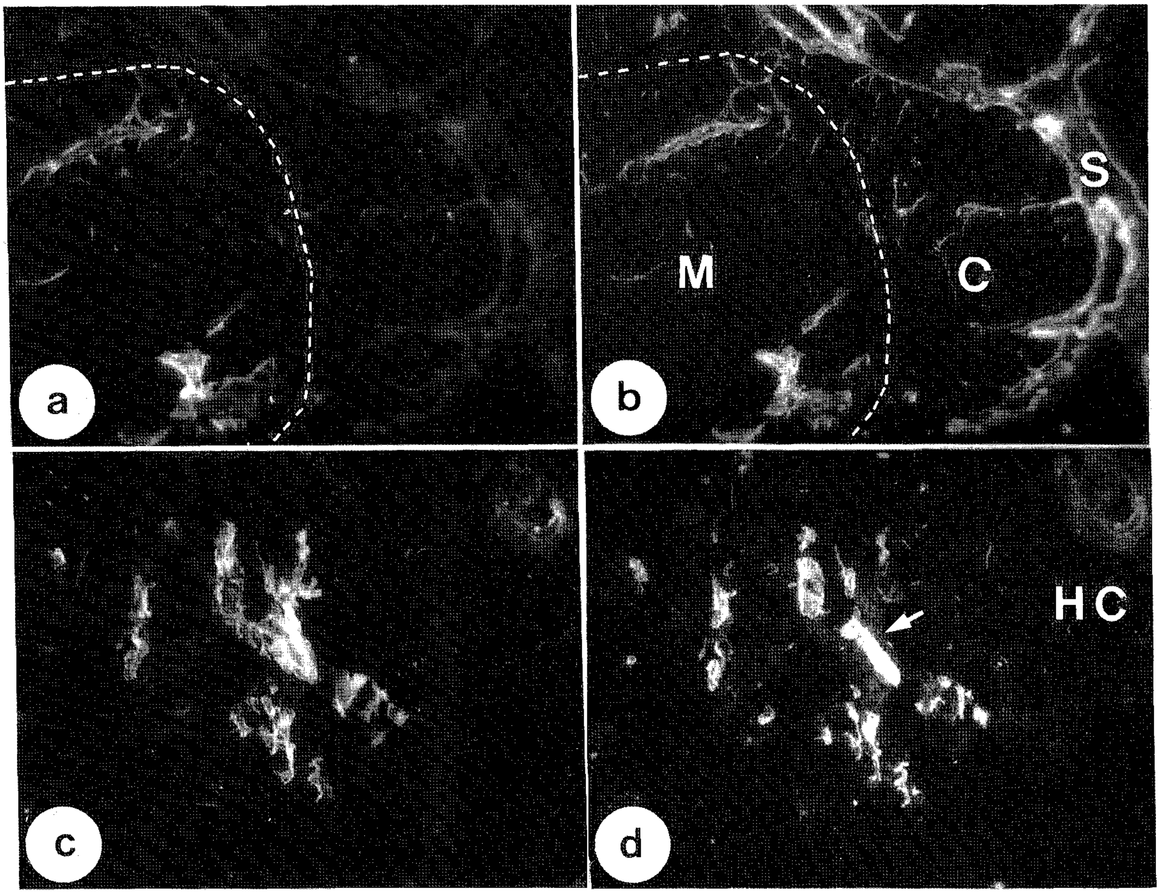

FIGURE 2. Partial co-localization of tenascin and other basement membrane proteins in the normal infant thymus. In panels (a) and (b), sections were double-labeled with anti-tenascin mAb and anti-type IV collagen serum, respectively. Note the septal basement membrane is type IV collagen-positive but TN-negative. In contrast, in the medullary region, the two proteins are virtually co-localized. Panels (c) and (d) depict a medullary area double-labeled for detection of tenascin (c) and laminin (d). The blood vessel shown in the center of the field (arrow) revealed that, although the basement membrane is double-positive for TN and LN, an external (adventitial) layer is only labeled for TN. Dashed lines represent the cortico-medullary limits. S: septum; HC: Hassall's corpuscle. Magnification: × 250.

network, although in the medulla, TN labeling could be detected adjacently to epithelial cells (Fig. 3).

### Expression of Tenascin by Cultured Thymic Microenvironmental Cells

We also investigated TN expression *in vitro*, using primary cultures developed from explants of normal infant thymuses. In this system, we found that CK (used as TEC marker) and TN labelings were mutually exclusive (Fig. 4). Moreover, the relatively few TN-bearing cells did not exhibit the typical fibroblast spindle-shaped profile. Yet, double-labeling experiments showed that TN-containing cells could also express laminin (Fig. 4). In this respect, extracellular fibers containing both laminin and TN could be observed.

### Expression of Tenascin in Thymus Ontogeny

Fetal thymus specimens with 12, 18, 25, and 30 weeks of gestational age were analyzed for tenascin expression and distribution, in relationship to the epithelial network. At 12 weeks, intrathymic tenascin expression was apparently incipient, because a positive immunocytochemical signal only could be clearly detected by the use of a three-layer labeling. Moreover, the TN distribution pattern at this gestational age was peculiar, ocurring in both peripheral and central regions of the thymic lobules. From the

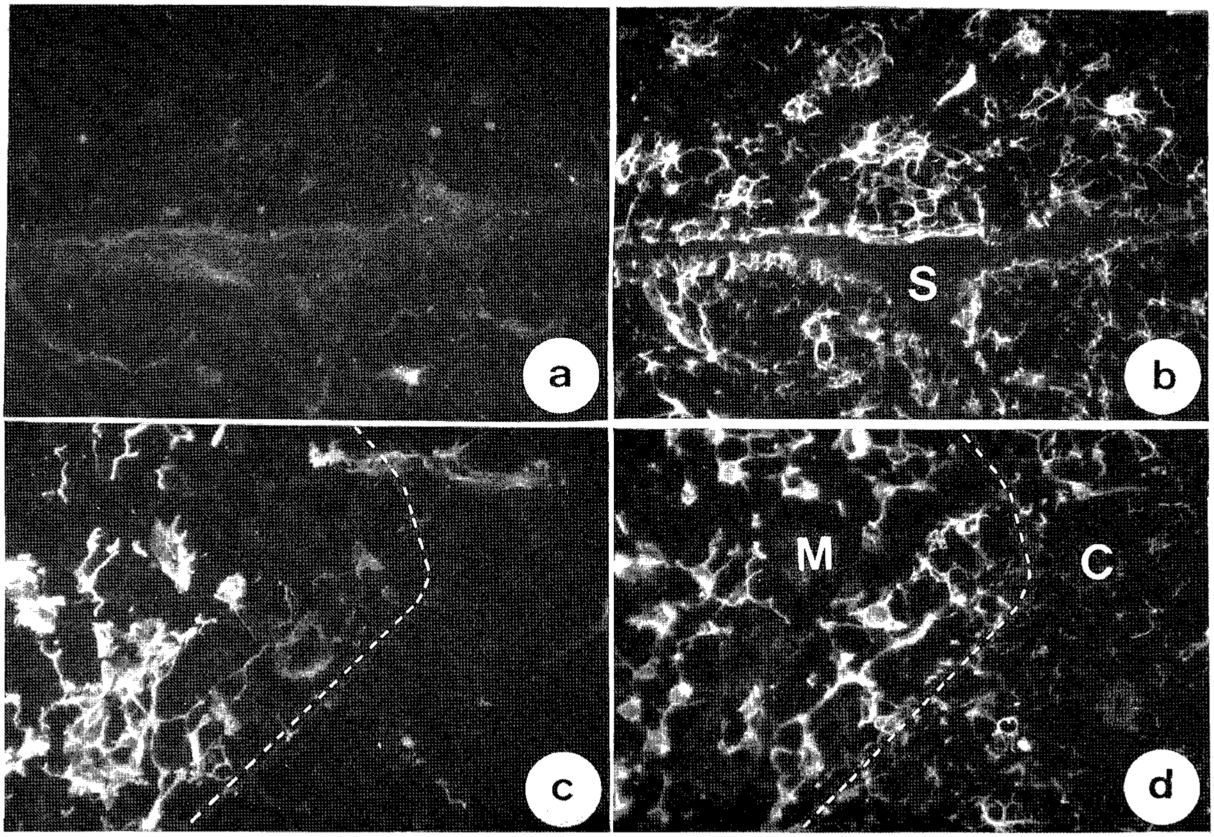

FIGURE 3. Tenascin does not co-localize with epithelial (CK-positive) cells in normal infant thymus. Sections were double-labeled with anti-tenascin polyclonal serum [panels (a) and (c)] and the anti-cytokeratin mAb KL1 [(b) and (d)]. Note that in the cortex, TN is absent in the epithelial network, and within the medulla, the TN-bearing network is rather adjacent to the epithelial cells. Dashed lines represent the cortico-medullary junction. S: septum; C: cortex; M: medulla. Magnification: ×250

18th week forward, the TN network was strongly labeled (even using a two-layer labeling), and the medullary-restricted topography already could be evidenced, similarly to what was observed in infant thymuses (Fig. 5).

A further aspect, which can be observed in Fig. 6, was that even in fetal stages during thymus ontogeny, TN staining was seen adjacently to the epithelial network, but never superposed to it.

### DISCUSSION

The present work represents an immunocytochemical survey regarding the localization of tenascin in the human thymus.

One relevant tissue concerning our study is that TN expression already could be detected in 12-week thymus rudiments, remaining constitutively in normal infant, as well as adult MG-associated hyperplastic organs (not shown). Nevertheless, because in early stages of thymus ontogeny TN labeling was incipient, it is possible that the expression of this molecule in the human thymus is developmentally upregulated. Such possibility is further supported by the differential topography of TN labeling in 12-week fetal thymus, comprising peripheral and central areas of thymic lobules.

In any case, our findings are in keeping with recent data revealing mRNA transcripts for TN in developing and adult murine thymuses (Saga et al., 1991; Ocklind et al., 1993), and places the thymus as one of the few organs in which TN expression is not down-regulated after fetal life. Interestingly, this seems to be a general feature in the immune system, because TN was also detected in spleen and lymph nodes (Liakka and Autio-Harmainen 1992; Ocklind et al., 1993). Taking into account that TN is apparently involved in regulating transient lymphocyte adhesion events (Ruegg et al., 1989), it is possible

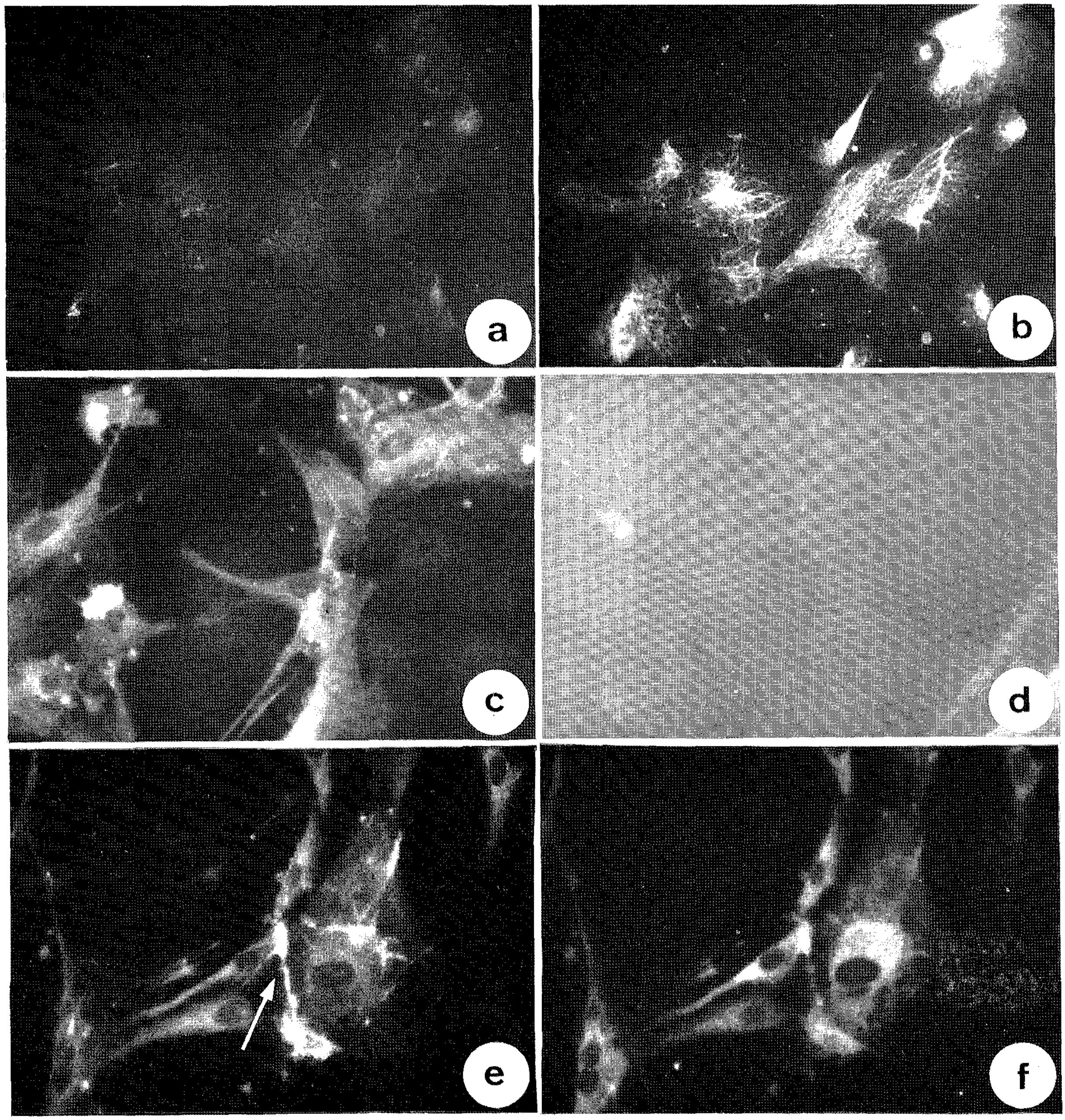

FIGURE 4. Expression of tenascin by cultured human thymic microenvironmental cells. Cell layers were double-labeled for detection of tenascin [panels (a), (c), and (e)] and cytokeratin [panels (b) and (d) or laminin [panel (f)]. Note the mutual exclusive labeling for TN and CK. Differently, TN-bearing cells also produce laminin. A TN/LN double-positive extracellular fiber can actually be seen (arrow). Magnification: ×250.

that TN plays a physiological role in lymphocyte migration, in both central and peripheral lymphoid organs. Should it be the case, one could predict that a similar cell would be responsible for TN synthesis in the various lymphoid organs. Yet, Ocklind et al. (1993) recently suggested that in mice, epithelial cells (which are restricted to the thymus, not existing in peripheral lymphoid organs) would be responsible for TN expression. However, our data concerning double-labeling immunofluorescence performed *in situ* and *in vitro* virtually discarded the hypothesis raised by those authors. One might

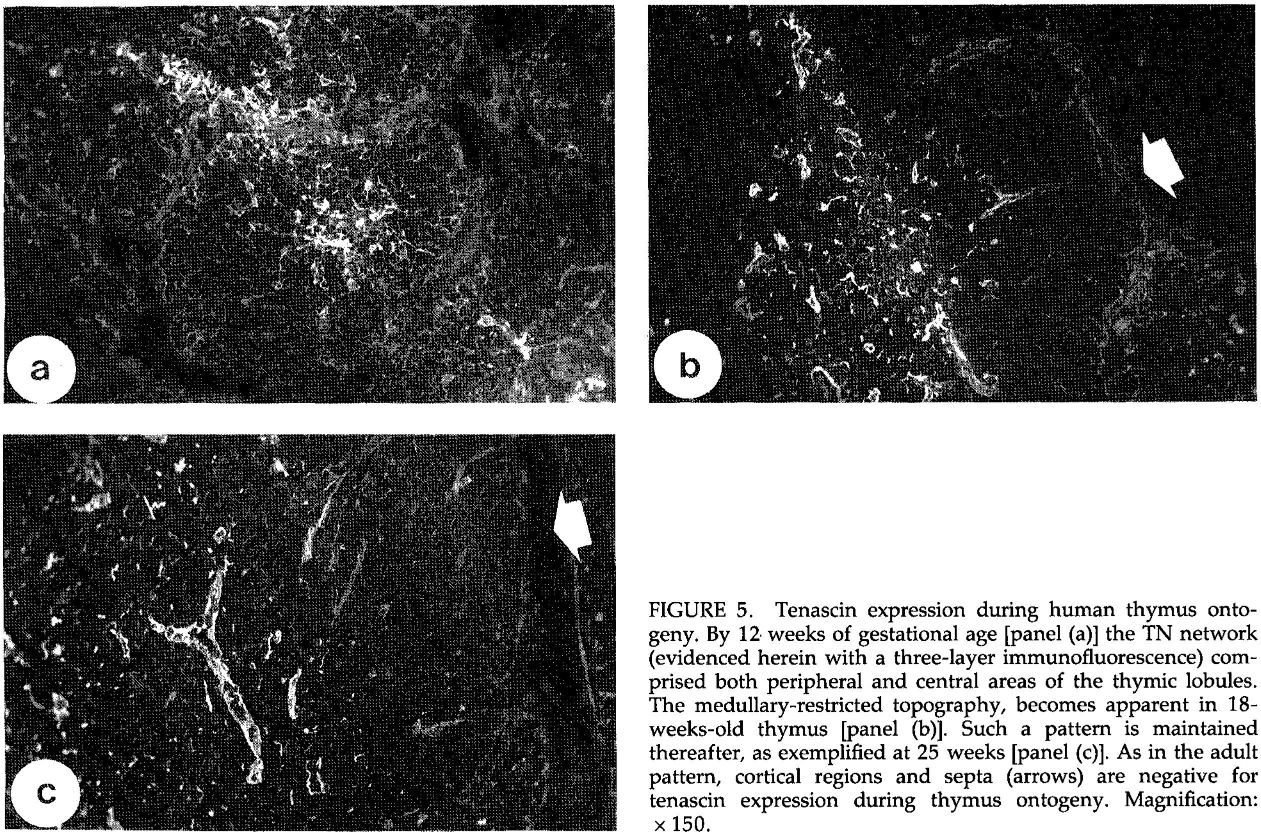

FIGURE 5. Tenascin expression during human thymus ontogeny. By 12 weeks of gestational age [panel (a)] the TN network (evidenced herein with a three-layer immunofluorescence) comprised both peripheral and central areas of the thymic lobules. The medullary-restricted topography, becomes apparent in 18-weeks-old thymus [panel (b)]. Such a pattern is maintained thereafter, as exemplified at 25 weeks [panel (c)]. As in the adult pattern, cortical regions and septa (arrows) are negative for tenascin expression during thymus ontogeny. Magnification: ×150.

argue that in those studies, the species analyzed was not the same. Nonetheless, we obtained similar results in mice (unpublished observations), which is in keeping with the phylogenetic conservation of extracellular matrix components in respect to their intrathymic distribution (Berrih et al., 1985; Lannes-Vieira et al., 1991; Meireles de Souza et al., 1993).

Additionally, typical fibroblasts do not seem to secrete tenascin in the thymus. This is supported by the virtual absence of TN within the thymic septa (which contain fibroblasts), and by the negative staining observed in spindle-shaped cells that grow in primary culture of human thymic microenvironmental cells, and that can even form *in vitro* septumlike multicellular structures (not shown).

A reticular cell of mesenchymal origin thus could be incriminated as the source of tenascin, both intrathymically and in the peripheral lymphoid organs. In fact, the perivascular and reticular TN distribution within the thymus and in both lymph node and spleen would fit with this possibility. Moreoever, *in vitro*, these TN-bearing cells also produce laminin, and the two ECM proteins can be found in the same extracellular fiber. Thus, a pericyte might be the source of tenascin within the immune system. Further work will hopefully test this possibility.

Lastly, it remains to be determined the role of tenascin in the physiology of the thymus and peripheral lymphoid organs. By taking into account its topography, adjacently to blood vessels and migrating cells, it is feasible to conceive that tenascin may be implicated in the exit of the lymphocytes from these organs, a hypothesis that is presently under investigation.

### ACKNOWLEDGMENTS

The authors thank Dr. Ivanir M. Oliveira (Miguel Couto Hospital, Rio de Janeiro) for the fetal thymuses; Dr. Ruth Chiquet-Ehrismann for kindly providing one anti-tenascin polyclonal antibody, and to Genilton J. Vieira for photographic technical support. This work was partially supported with grants from CNPq/PADCT (Brazil), the National Cancer Institute (Brazil), and CEC (Brussels).

*(Received May 16, 1994)*

*(Accepted May 31, 1994)*

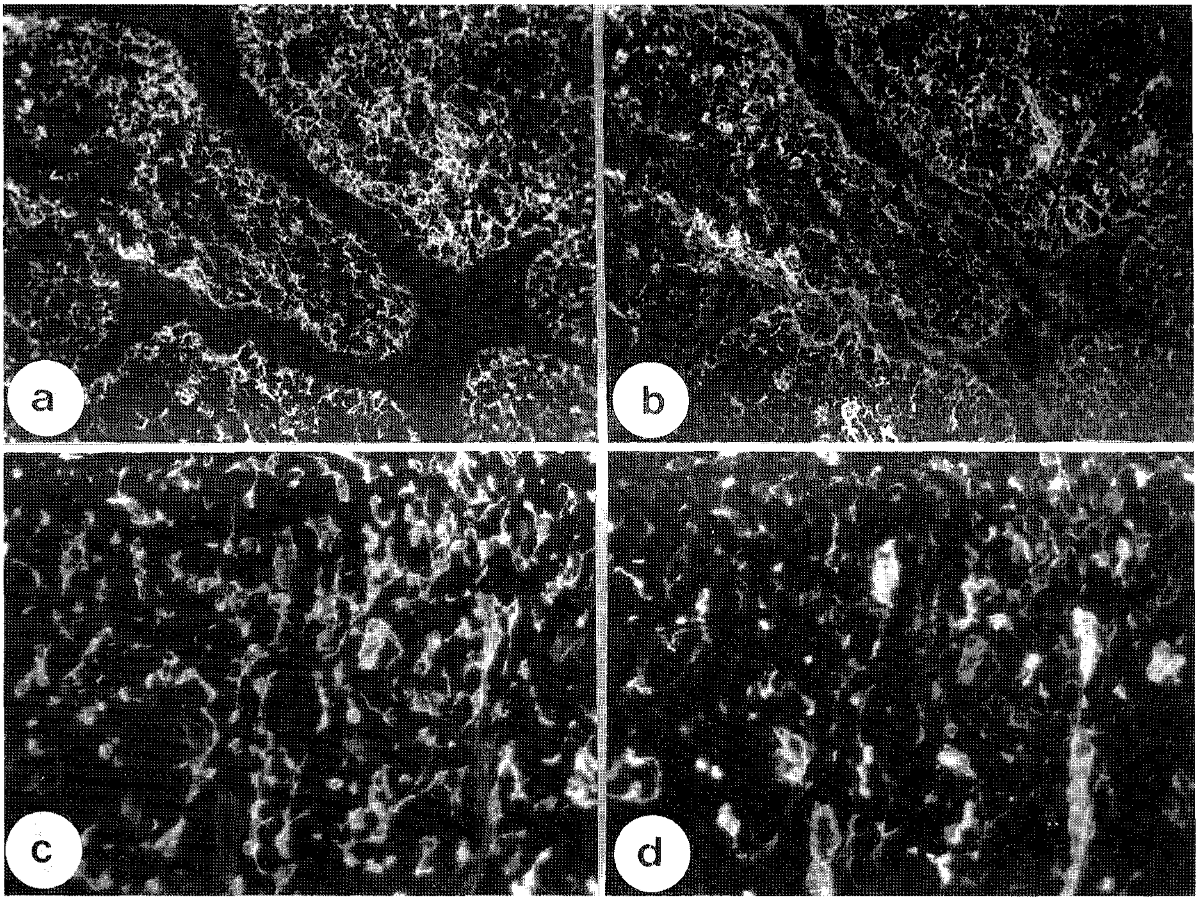

FIGURE 6. Tenascin does not co-localize with epithelial (CK-positive cells) in fetal thymuses. Panels (a, c) and (b, d) depict respectively, anti-cytokeratin (mAb KL4) and anti-TN in 12-week-old thymus. In panels (c) and (d), a medullary region is seen in a 18-week-old thymus with TN staining being seen adjacent to epithelial cells, apparently surrounding vessels. Magnifications: × 150 and × 250, respectively, for panels (a, b) and (c, d).

### REFERENCES

Aufderheide E., and Ekblom P. (1988). Tenascin during gut development: appearance in the mesenchyme, shift in molecular forms, and dependence on epithelial-mesenchymal interactions. J. Cell Biol. **107**: 2341–2349.

Berrih S., Savino W., Azoulay M., Dardenne M., and Bach J.F. (1984). Production of anti-thymulin (FTS) monoclonal antibodies by immunization against human thymic epithelial cells. J. Histochem. Cytochem. **32**: 432–438.

Berrih S., Savino W., and Cohen S. (1985). Extracellular matrix of the human thymus: Immunofluorescence studies on frozen sections and cultured epithelial cells. J. Histochem. Cytochem. **33**: 655–664.

Boyd R., and Hugo P. (1991). An integrated view of the thymic microenvironment. Immunol. Today **132**: 718–720.

Boyd R.L., Tucek C.L., Godfrey D.I., Izon D.J., Wilson T.J., Davidson N.J., Bean A.G.D., Ladyman H.M., Ritter M.A., and Hugo P. (1993). The thymic microenvironment. Immunol. Today **14**: 421–427.

Chiquet M., and Fambrough D.M. (1984a). Chick myotendinous antigen. I. A monoclonal antibody as a marker for tendon and muscle morphogenesis. J. Cell Biol. **98**: 1926–1936.

Chiquet M., and Fambrough D.M. (1984b). Chick myotendinous antigen. II. A novel extracellular glycoprotein complex consisting of large disulfide-linked subunits. J. Cell Biol. **98**: 1937–1946.

Chiquet-Ehrismann R. (1990). What distinguishes tenascin from fibronectin? FASEB J. **4**: 2598–2604.

Chiquet-Ehrismann R., Kalla P., Pearson C.A., Beck K., and Chiquet M. (1988). Tenascin interferes with fibronectin action. Cell **53**: 383–390.

Crossin K.L., Hoffmann S., Grumet M., Thiery J.-P., and Edelman G.M. (1986). Site-restricted expression of cytotactin during development of the chicken embryo. J. Cell Biol. **102**: 1917–1930.

Donaldson D.J., Mahan J.T., Yang H., and Crossin K.L. (1991). Tenascin localization in skin wounds of the adult newt Notophtalmus viridescens. Anat. Record **230**: 451–459.

Grimaud J.A., Druguet M., Peyrol S., Chevalier O., Herbage D., and El-Badravoi, N. (1980). Collagen immunotyping in human liver. J. Histochem. Cytochem. **28**: 1145–1156.

Howeedy A.A., Virtanen I., Laitanen L., Gould N.S., Koukoulis G.K., and Gould, V.E. (1990). Differential distribution of tenascin in the normal, hyperplastic, and neoplastic breast. Lab. Invest. **63**: 798–806.

Inaguma Y., Kusakabe M., Mackie E.J., Pearson C.A., Chiquet-Ehrismann R., and Sakakura T. (1988). Epithelial induction of stromal tenascin in the mouse mammary gland: From embryogenesis to carcinogenesis. Dev. Biol. **128**: 245–255.

Jones F.S., Hoffmann S., Cunningham B.A., and Edelman, G.M. (1989). A detailed structural model of cytotactin: Protein homologies, alternative RNA splicing, and binding regions. Proc. Natl. Acad. Sci. USA **86**: 1905–1909.

Lannes-Viera J., Dardenne M., and Savino W. (1991). Extracellular matrix components of the mouse thymus microenvironment: Ontogenetic studies and modulation by glucocorticoid hormones. J. Histochem. Cytochem. **39**: 1539–1546.

Liakka K.A., and Autio-Harmainen H.I. (1992). Distribution of the extracellular matrix proteins tenascin, fibronectin, and vitronectin in fetal, infant, and adult human spleens. J. Histochem. Cytochem. **40**: 1203–1210.

Lotz M.M., Burdsal C.A., Erickson H.P., and McClay D.R. (1989). Cell adhesion to fibronectin and tenascin: Quantitative measurements of initial binding and subsequent strengthening response. J. Cell Biol. **109**: 1795–1805.

Mackie E.J., Chiquet-Ehrismann R., Pearson C.A., Inaguma Y., Taya K., Kawarada Y., and Sakakura T. (1987). Tenascin is a stromal marker for epithelial malignancy in the mammary gland. Proc. Natl. Acad. Sci. USA **84**: 4621–4625.

Mackie E.J., Halfter W., and Liverani, D. (1988). Induction of tenascin in healing wounds. J. Cell. Biol. **107**: 2577–2767.

Meireles de Souza L.R., Trajano V., and Savino W. (1993). Is there an interspecific diversity of the thymic microenvironment? Dev. Immunol. **3**: 123–125.

Natali P.G., Nicotra M.R., Bigotti A., Botti C., Castellani P., Risso A.M., and Zardi, L. (1991). Comparative analysis of the expression of the extracellular matrix protein tenascin in the normal human fetal, adult and tumor tissues. Int. J. Cancer **47**: 811–816.

Ocklind G., Talts J., Fassler R., Mattsson A., and Ekblom P. (1993). Expression of tenascin in developing and adult mouse lymphoid organs. J. Histochem. Cytochem. **41**: 1163–1169.

Papiernik M., Nabarra B., and Bach J.F. (1975). *In vitro* culture of functional human thymic epithelium. Clin. Exp. Immunol. **19**: 281–287.

Prieto A.L., Jones F.S., Cunningham B.A., Crossin K.L., and Edelman G.M. (1990). Localization during development of alternatively spliced forms of cytotactin mRNA by in situ hybridization. J. Cell Biol. **111**: 685–698.

Ruegg C.R., Chiquet-Ehrismann R., and Alkana S.S. (1989). Tenascin, an extracellular matrix protein, exerts immunomodulatory activities. Proc. Natl. Acad. Sci. USA **86**: 7437–7441.

Saga Y., Tsukamoto T., Jing N., Kusakabe M., and Sakakura T. (1991). Murine tenascin: cDNA cloning, structure and temporal expression of isoforms. Gene **104**: 177–185.

Savino W., and Berrih S. (1984). Thymic extracellular matrix in myasthenia gravis. Lancet **2**: 45–46.

Savino W., Villa-Verde D.M.S., and Lannes-Vieira J. (1993). Extracellular matrix proteins in intrathymic T-cell migration and differentiation? Immunol. Today **14**: 158–161.

Soini Y., Alavaikko M., and Lehto V.-P. (1992b). Tenascin in reactive lymph nodes and in malignant lymphomas. Path. Res. Pract. **188**: 1078–1082.

Soini Y., Paakko P., Virtanen I., and Lehto V.-P. (1992a). Tenascin in salivary gland tumours. Virch. Arch. Path. Anat. **421**: 217–222.

Spring J., Beck K., and Chiquet-Ehrismann R. (1989). Two contrary functions of tenascin: Dissection of the active sites by recombinant tenascin fragments. Cell **59**: 325–333.

Thesleff I., Vainio S., Salmivirta M., and Jalkanen M. (1990). Syndecan and tenascin: Induction during early tooth morphogenesis and possible interactions. Cell Diff. Devl. **32**: 383–390.

Van Ewijk W. (1991). T-cell differentiation is influenced by thymic microenvironments. Ann. Rev. Immunol. **9**: 591–615.

